# Multispectral Optoacoustic Tomography of Skeletal Muscle Unveils Microcirculation and Oxygen Metabolism Alterations in Sarcopenia

**DOI:** 10.1002/jcsm.70088

**Published:** 2025-10-21

**Authors:** Ying Yang, Dan Wu, Yunqing Xv, Yonghua Xie, Xinsheng Wang, Yuanyuan Bi, Jing Zhang, Yun Wu, Yanting Wen, Shixie Jiang, Yiran Zhang, Tangming Peng, Zheng Li, Jiehao Chen, Xiaoyan Chen, Binglong Wang, Shanping Chen, Ming Yang, Huabei Jiang

**Affiliations:** ^1^ School of Computer Science and Technology Chongqing University of Posts and Telecommunications Chongqing China; ^2^ Geriatric Diseases Institute of Chengdu/Cancer Prevention and Treatment Institute of Chengdu, Department of Neurology Chengdu Fifth People's Hospital Chengdu China; ^3^ Department of Ultrasound Medicine Chengdu Fifth People's Hospital Chengdu China; ^4^ Department of Psychiatry University of Florida College of Medicine Gainesville Florida USA; ^5^ Department of Pathology Department Chengdu Fifth People's Hospital Chengdu China; ^6^ Animal Laboratory Center, West China Hospital Sichuan University Chengdu China; ^7^ Department of Geriatrics Zigong Affiliated Hospital of Southwest Medical University Zigong China; ^8^ Department of Geriatrics Chengdu Fifth People's Hospital Chengdu China; ^9^ Center of Gerontology and Geriatrics, West China Hospital Sichuan University Chengdu China; ^10^ Department of Medical Engineering University of South Florida Tampa Florida USA

**Keywords:** collagen, hemodynamics, image reconstruction, imaging, multispectral optoacoustic tomography, sarcopenia

## Abstract

**Background:**

Sarcopenia, a significant geriatric syndrome, faces challenges in accurate diagnosis due to limitations of current imaging techniques. This study explores the novel application of multispectral optoacoustic tomography (MSOT) in evaluating sarcopenia, focusing on quantifying oxygen dynamics and collagen distribution in skeletal muscles.

**Methods:**

We conducted MSOT imaging on the lower limbs of senescence‐accelerated mouse prone 8 (SAMP8; *n* = 14) and senescence‐accelerated mouse resistant 1 (SAMR1; *n* = 8) models, using light wavelengths of 760, 840 and 930 nm. CT, histopathology and immunofluorescence were used for cross‐validation.

**Results:**

Label‐free MSOT imaging directly visualized muscle structure and metabolism with high spatiotemporal resolution. Compared to SAMR1 controls, sarcopenic SAMP8 mice demonstrated 23.8% lower HbO_2_ levels (SAMP8: 0.0016 ± 0.0003 a.u. vs. SAMR1: 0.0021 ± 0.0005 a.u.; *p* = 0.018) and reduced metabolic activity in skeletal muscles. SAMP8 mice also revealed 43.2% higher collagen content (SAMP8: 3.451 ± 1.159 a.u. vs. SAMR1: 2.409 ± 0.635 a.u.; *p* = 0.030) alongside more disordered muscle structure, suggesting increased fibrosis. An inverse correlation was observed between computed tomography (CT) values and MSOT‐derived collagen signals (*r* = −0.789, *p* < 0.001), whereas no such correlation existed with HbO_2_, indicating that MSOT provides unique metabolic insights beyond traditional imaging techniques.

**Conclusions:**

This first application of MSOT in sarcopenia research highlights its potential as a noninvasive, real‐time tool for early diagnosis, therapeutic evaluation and mechanistic understanding. Its ability to detect metabolic changes not captured by CT underscores its complementary role in comprehensive muscle assessment. Future research should focus on longitudinal studies and clinical translation.

## 1 Introduction

Sarcopenia, a progressive skeletal muscle disorder characterized by loss of muscle mass and strength, significantly increases the risk of adverse outcomes such as falls, functional decline, frailty and mortality [1, 2, 3]. Recent epidemiological studies have revealed a concerning prevalence of sarcopenia, ranging from 8% to 36% in nonaged individuals and 10% to 27% in those over 60 years of age [2, S1]. Given its substantial impact on individual health and healthcare systems, the European Working Group on Sarcopenia in Older People (EWGSOP) has emphasized the critical importance of early diagnosis and intervention to prevent or delay adverse health outcomes [4].

Current diagnostic approaches for sarcopenia primarily focus on assessing muscle mass and muscle strength, typically employing a combination of imaging techniques and functional tests [5, 6, 7, 8, 9, S2]. Existing imaging methods for quantifying muscle mass face significant limitations, creating a bottleneck in accurate diagnosis and assessment. Conventional radiological techniques such as dual‐ energy x‐ray absorptiometry (DXA) lack specificity for muscle tissue and can be influenced by hydration status [7, 8, 10, 11]. While computed tomography (CT) and magnetic resonance imaging (MRI) are considered gold standards for evaluating muscle mass, they are limited by high cost, complexity and, in the case of CT, radiation exposure [12, 13, S2]. Ultrasound (US), although portable and radiation‐free, lacks standardized cutoff values and suffers from poor reproducibility across different operators [9, 14].

Multispectral optoacoustic tomography (MSOT) emerges as a promising solution to these diagnostic challenges. This innovative, noninvasive imaging technology combines light and sound to provide in vivo images with submillimetre spatial resolution and centimetre‐level tissue penetration [15]. For sarcopenia assessment, MSOT simultaneously images muscle structure and function. It provides high‐resolution vascular structural data and quantifies molecules (haemoglobin, collagen) via optical absorption differences, enabling metabolic capacity evaluation [16]. This elucidates sarcopenia pathophysiology at macroscopic and microscopic scales [17, S3, S4]. MSOT requires no ionizing radiation, typically eliminates the need for exogenous contrast agents, and is highly cost‐effective, with costs generally 1 to 2 orders of magnitude lower than common imaging modalities such as CT and MRI, while maintaining comparable diagnostic efficacy for specific applications [18, 19, 20].

In this pioneering study, we aimed to explore the application of MSOT in sarcopenia research. Our primary objectives were to investigate MSOT's potential for real‐time, label‐free imaging of oxyhemoglobin (HbO_2_), deoxyhemoglobin (HbR), oxygen saturation (sO_2_) and collagen in sarcopenic mice and characterize muscle function and metabolism using these MSOT‐derived biomarkers. Secondary objectives included evaluating hemodynamic changes and quantifying multiple hemodynamic parameters in muscle tissue, along with cross‐validating MSOT measurements against CT and histopathological evaluation to ensure reliability and accuracy. We utilized two established mouse models relevant to human aging: the senescence‐accelerated mouse prone 8 (SAMP8), which exhibits accelerated aging, a shortened lifespan and earlier onset of age‐related pathologies like sarcopenia at approximately twice the rate of normal aging and the senescence‐accelerated mouse resistant 1 (SAMR1), which serves as the corresponding control strain aging at a normal rate [1, 21]. High‐resolution MSOT images of SAMP8 and SAMR1 mice were acquired and analyzed separately.

## 2 Materials and Methods

### 2.1 MSOT Imaging System

**FIGURE 1:**
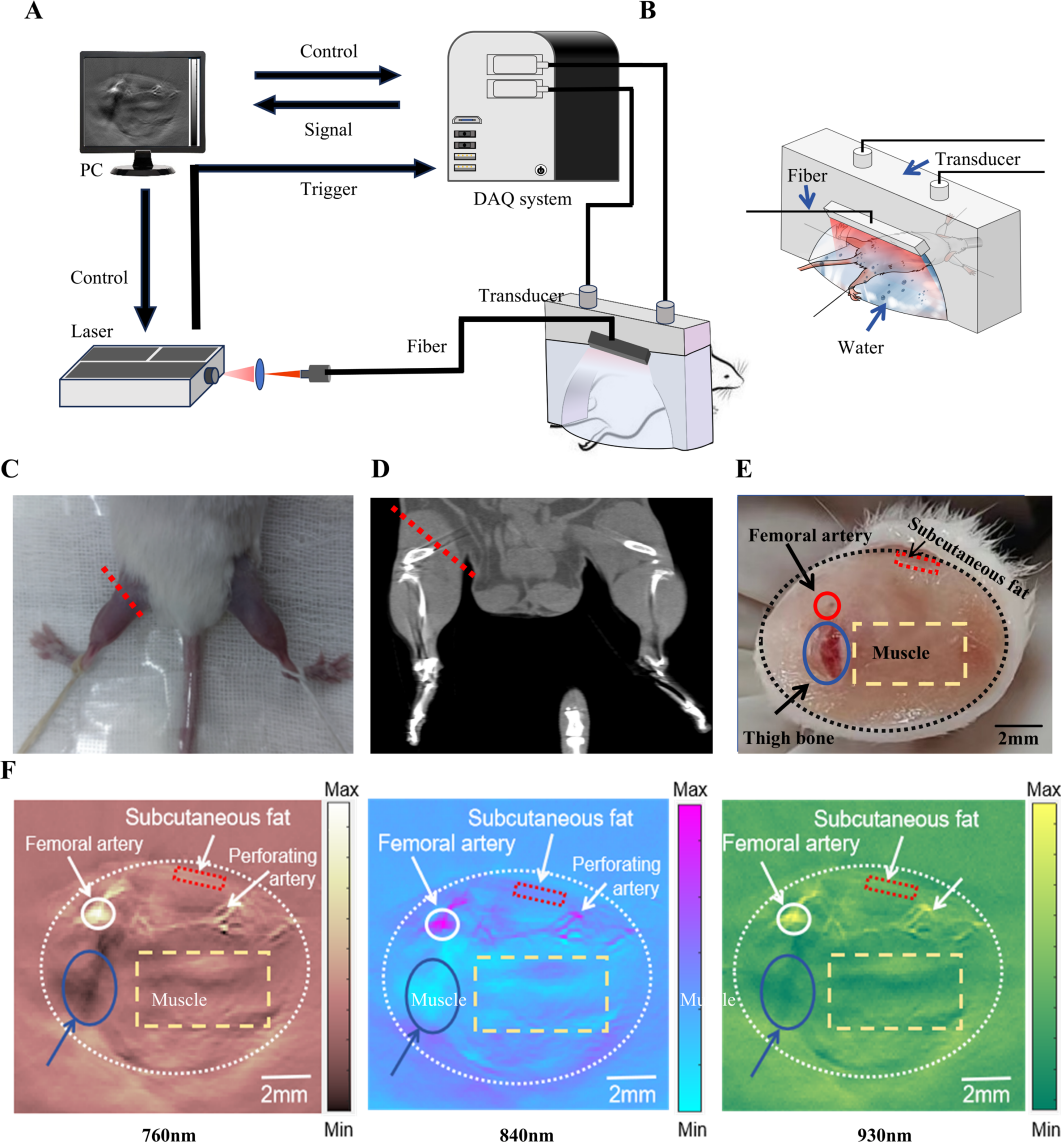
A schematic of the MSOT system for mouse muscle imaging. B. Simulation diagram of MSOT data collection from the lower limb muscles of mice. Remove the leg hair from the lower limbs of mice, clean with depilatory cream, and apply an ultrasonic coupling agent to maximize ultrasonic transmission. The picture showed the position of the mouse and probe. C. Demonstration of the anatomical location of MSOT imaging on live mice. D. Demonstration of the anatomical location of MSOT imaging on CT image. E. Cross‐sectional anatomical diagram of the mouse lower limb at the corresponding level of MSOT imaging. F. From left to right, respectively displayed the MSOT imaging at different wavelengths of 760, 840 and 930 nm. DAQ, computer, data acquisition; PC, personal computer; US, ultrasound.

The MSOT system used in this study is shown in Figure 1A. In this system, excitation source was provided by a high‐frequency OPO pulsed laser (SpitLight EVO S OPO, Innolas), with a pulse width of 5 ns and a pulse repetition frequency of 100 Hz. The laser beam, focused by a convex lens, was directed onto the biological tissue via an optical fibre. US gel was applied to enhance ultrasonic coupling. Photoacoustic signals were received by a custom‐made 256‐channel semicircular ultrasonic transducer (centre frequency: 5 MHz, curvature radius: 80 mm). These signals were then collected and processed through a custom 256‐channel real‐time data acquisition card (Marsonics DAQ 256, Marsonic, Tianjin, China), with a sampling rate of 40 MSPS, a resolution of 14 bits and a maximum gain of 80 dB. The system achieved a temporal resolution of 100 μs and a spatial resolution of 100 μm. In the current configuration, the laser system's penetration depth exceeded 3 cm, sufficient to image subcutaneous fat and superficial muscle tissue in mouse lower limbs, while also generating reflection US CT mode images.

### 2.2 Animals

SAMP8 (P8) and SAMR1 (R1) were obtained from the Department of Laboratory Animal Science, Peking University Health Science Center (Permit Number: SCXK‐2021‐0013). Healthy 32‐week‐old male specific‐pathogen‐free (SPF) P8 and R1 mice (weight: 25–35 g) were housed in pairs under controlled conditions (temperature: 20–22 °C, relative humidity: 40%–60%, 12 h light–dark cycle) with ad libitum access to distilled water and standard chow. Mice underwent a 7‐day acclimation period before photoacoustic imaging. Two groups were established: (A) P8 (*n* = 14) and (B) R1 (*n* = 8). A validation cohort (*n* = 4 for P8 group, *n* = 4 for R1 group) was added during revision to obtain better histological results. To minimize movement during data collection, mice were anaesthetized with isoflurane immediately before imaging and positioned on a brain stereotaxic system (68 001, RWD Life Science, China). Leg hair was removed using depilatory cream, and US coupling gel was applied to optimize the US transmission (Figure 1B).

### 2.3 MSOT Image Reconstruction

The MSOT system described above was used to process data from the thickest part of each mouse's left lower limb, designated as the region of interest (ROI). To ensure consistency in MSOT data collection, we identified the cross‐sectional area (CSA) where femoral artery perforators were clearly visible as the target level. The ROI acquisition image layer position is shown in Figure 1C and D. Tissue illumination was achieved using a periodic pattern of three different wavelengths (760, 840 and 930 nm) with short pulses (< 10‐ns duration) at a rate of 100 Hz. The multispectral reconstruction algorithm proposed by Jiang et al. was employed to analyze HbO_2_, HbR, sO_2_ and collagen content (Supplementary Figures 1, 2 and Supplementary Explanation 1) [19, 22, S5]. Figures 1F displayed the MSOT imaging at different wavelengths of 760, 840 and 930 nm, respectively. The reconstructed MSOT images corresponded well with the anatomical structure observed in the actual cross‐sectional image of the mouse leg (Figure 1E).

### 2.4 Micro‐CT

Micro‐CT was performed on isoflurane‐anaesthetized mice in prone position 3 days prior to our experiment to assess muscle density and volume in the lower limbs. We maintained consistent positioning and posture for each mouse. Scanning was conducted using a NEMO Micro CT NMC‐100 (Pingsheng Medical Technology Kunshan Co. Ltd., China) with the following parameters: 0.1‐mm resolution, 60‐kV source voltage, 100‐mm lateral field of view (FOV), 35‐mm axial FOV and 0.13‐mA current. The x‐ray source rotation step was set on 0.7. The average scanning time per animal was approximately 5 min. Reconstructed images were analyzed using AVATAR software (Version 1.6.9.3, Pingsheng Medical Technology Co. Ltd., Kunshan, China) employing an iterative reconstruction algorithm for 3D reconstruction. The reconstructed 3D image had a size of 0.1 × 0.1 × 0.1 mm. A single slice‐based analysis was performed, with the volume (mm^3^) and density (in Hounsfield units, Hu) of the ROI muscle, designated as ‘lower limb muscle.’ measured semiautomatically. The ROI selection and delineation are illustrated in Supplementary Figure 3.

### 2.5 Muscle Strength and Rotarod Tests

Muscle strength and rotarod tests were conducted 3 days after the MOST experiment. Muscle strength was measured using a force gauge (Yiyan Technology Development Co. Ltd., Jinan, China). Mice were made to grasp a horizontal rod using their forelimbs while their tails were restrained to prevent hindlimb use. The rod was slowly pulled until the mice released their grip. The maximum grip strength from five trials was recorded, with the average value considered as the mouse's muscle strength. Grip strength was measured in gram force (gf) [21].

An accelerating rotarod device (Yiyan Technology Development Co. Ltd., Jinan, China) was used to assess motor function coordination and balance. Mice underwent a 3‐day training period before formal data collection. The device's acceleration increased from 4 to 40 rpm over a 5‐min period. The time spent on the rod (latency to fall) was recorded each trial, with three testing periods per mouse. We analyzed the longest duration time. The rotarod test results were recorded in seconds (s) [23].

### 2.6 Histopathological Analysis

#### 2.6.1 H&E Histology

Muscle samples obtained from the gastrocnemius were cryosectioned for histological examination. The samples underwent fixation, dehydration, embedding, slicing and sectioning before being mounted on glass slides. Prior to staining, xylene was used to remove the embedding medium. Muscle sections of 5‐μm thickness (Cryostar NX70, Thermos Scientific, MA, USA) were placed on silane‐coated glass slides and subjected to H&E staining to evaluate muscle tissue morphology [21, S6, S7].

#### 2.6.2 Immunohistochemistry

Immunofluorescence staining of myosin heavy chain (MHC) isoforms was performed to characterize muscle fibre types, following the protocol described by Chen et al. [24] A primary antibody cocktail was prepared by mixing antibodies against MHC I (BA‐D5, DSHB), MHC IIa (SC‐71, DSHB) and MHC IIb (BF‐F3, DSHB). Following several washes, sections were incubated with Alexa Fluor‐conjugated secondary antibodies (647, 488, 555) in Quick Block buffer for 2 h at room temperature (RT). Images were captured using a Nikon C2 microscope. After image acquisition (DM6000B, Leica, Wetzlar, Germany), the areas corresponding to MHC I (green), MHC lla (blue) and MHC llb (red) were visualized and merged using the ImageJ software (NIH, Bethesda, USA).

Immunohistochemical staining for collagen I and III was performed as follows: Sections were deparaffinized in xylene, rehydrated through a graded ethanol series, and rinsed in distilled water (5 min). Antigen retrieval was conducted in citrate buffer (pH 6.0). After blocking endogenous peroxidase with 3% H_2_O_2_ (15 min, RT, dark), slides were washed three times in PBS (pH 7.4) for 5 min each on a rocker. Nonspecific binding was blocked with 5% normal goat serum (Biosharp, BL210A) in PBS (30 min, RT). Sections were incubated overnight at 4 °C with primary antibodies: collagen I (1:200, Proteintech, 14 695‐1‐AP) and collagen III (1:200, Affinity, AF5457). After three PBS washes, 100 μL of TSA working solution (prepared by mixing 1‐mL tyramide dilution buffer with 2 μL CY3‐tyramide) was applied per section. Following additional PBS washes, sections were incubated with goat anti‐rabbit IgG secondary antibody (1:200, MultiSciences, GAR0072) for 1 h at RT. After four final PBS washes, sections were mounted with DAPI‐containing antifade medium [S8].

#### 2.6.3 Western Blotting

Western blotting was performed to analyze collagen expression. Gastrocnemius muscle tissues were homogenized on ice in RIPA lysis buffer (Biosharp, BL504A) containing 1 mM PMSF and phosphatase inhibitors. Lysates were centrifuged at 12000 × g for 5 min at 4 °C, and supernatants were collected. Proteins were separated on 10% SDS–polyacrylamide gels and transferred to 0.45–μm PVDF membranes (Merck Millipore, IPVH00010) at 300 mA for 30 min using a wet‐transfer system. Membranes were blocked with 5% nonfat milk in TBST for 1 h at RT, followed by overnight incubation at 4°C with primary antibodies against: collagen I (1:2000, Proteintech, 14 695‐1‐AP), collagen III (1:1000, Affinity, AF5457) and β‐actin (1:5000, Affinity, AF7021). After washing, membranes were incubated with HRP‐conjugated goat anti‐rabbit IgG (1:10 000, MultiSciences, GAR0072) for 1 h at RT. Signals were developed using ECL substrate (Biosharp, BL523B), captured on a ChemiScope 6100 imaging system (Clinx, Shanghai), and quantified with ChemiScope Analysis software [S9].

For both H&E and immunofluorescence staining, images were acquired at an identical 20 × magnification with CaseViewer. Three images per gastrocnemius muscle were analyzed and more than 200 muscle fibres were measured (*n* = 4) [S10]. In H&E sections, muscle‐fibre CSA was calculated with the Cross‐Sectional Analyzer plugin in ImageJ, while for immunofluorescence, mean fluorescence intensity (MFI) was measured in ImageJ (Fiji ImageJ v1.54p) after applying an appropriate threshold [S11, S12].

## 3 Ethics

The animal experiments were conducted in accordance with the Animals in Research: Reporting In Vitro Experiments (ARRIVE) guidelines 2.0 [25] and approved by the Ethics Committee for Laboratory Animals of Chongqing University of Posts and Telecommunications, Chongqing, China (No. 202428).

## 4 Data Analysis and Statistics

Data were analyzed using MATLAB (Version R 2022b, The Math Works Inc., Natick, MA, USA). MSOT images of the mice lower limb revealed arteries, femur, subcutaneous fat and muscle tissue (Figure 1F–H). In Figure 1F, three regions of interest (ROIs) were defined: ROI 1, a yellow rectangle (6 × 3 mm) representing muscle tissue, avoiding bone and large blood vessels; ROI 2, a red rectangle (2 × 0.5 mm) parallel to the skin surface, representing subcutaneous fat tissue; and ROI 3, a circular area (radius 0.5 mm) cantered on the most prominent femoral artery MSOT signal. All measurements were averaged within their respective ROIs. Results are presented in arbitrary units (a.u.) (Supplementary Explanation 2).

Statistical analysis was performed using GraphPad Prism (version 9.0, GraphPad Software Inc. San Diego, CA, USA). Mean values and standard deviations were calculated for all parameters (HbR, HbO_2_, SO_2_ and collagen). Student's *t* test was employed for statistical comparisons, with *p* values < 0.05 considered statistically significant. Spearman's rank correlation coefficients were calculated using IBM SPSS Statistics (version 25, International Business Machines Corporation, Armonk, NY, USA) to assess observer variability. Intraclass correlation (IC) values were interpreted as follows: 0.41–0.60 moderate, 0.61–0.80 substantial and 0.81–1.00 excellent agreement [26].

## 5 Results

### 5.1 Imaging Phenotypes of Healthy and Diseased Muscles

Real‐time MSOT imaging, performed noninvasively and without contrast agents, enabled precise anatomical localization of mouse lower limb structures including skin, femoral arteries and muscles in reconstructed data. This was followed by quantification of spectral signals to measure HbO_2_, HbR, sO_2_ and collagen levels. The images revealed a strong signal layer from the skin, underlaid by a low‐signal layer of adipose tissue.

Beneath the subcutaneous fat, the MSOT image delineated the femoral artery, its branches and the muscle region. Our analysis focused on the femoral artery, muscle and subcutaneous adipose tissue of both mouse types (ROIs shown in Figures 1F), as these areas were crucial for evaluating oxygen metabolism indicators and collagen fibre parameters in sarcopenia models.

**FIGURE 2:**
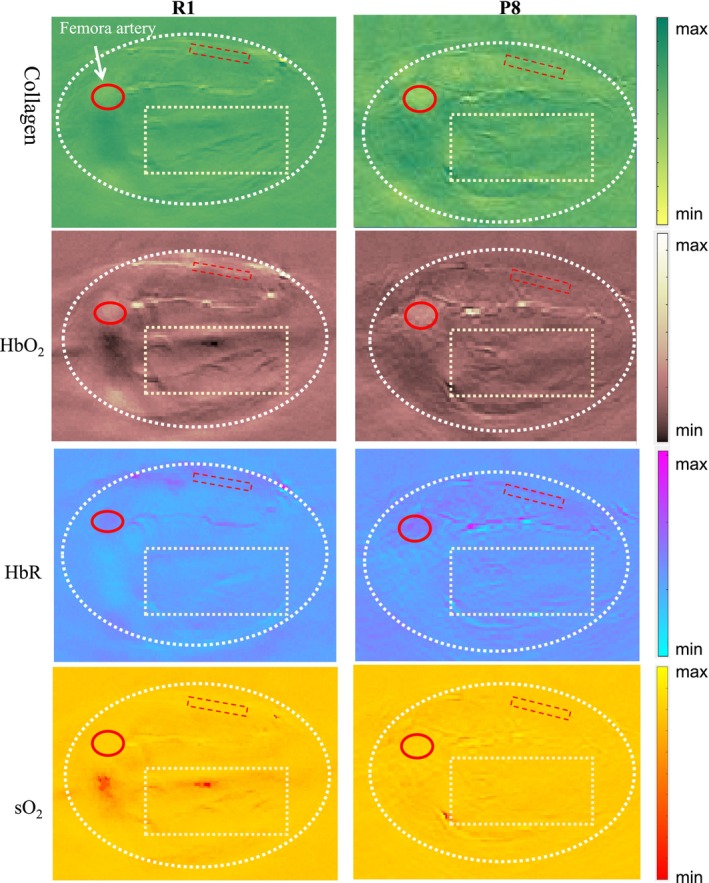
MSOT imaging of hind limbs: P8 vs. R1 mice: collagen, HbO_2_, HbR and sO_2_ (top to bottom). ROIs: (1) muscle tissue (yellow rectangle, 6 × 3 mm; ROI 1, excludes bone/large vessels), (2) subcutaneous fat (red rectangle, 2 × 0.5 mm; ROI 2) and (3) femoral artery (circle, radius 0.5 mm; ROI 3, cantered on maximal MSOT signal). Data are in arbitrary units (a.u.).

The P8 mice exhibited lower HbO_2_ and collagen signals compared to the R1 mice, particularly in the muscle area (Figures 2). R1 mice muscles exhibited a uniform distribution of blood vessels and well‐defined muscle fibres, whereas P8 mice muscles showed irregular vascular patterns and disorganized muscle fibres. No significant difference in sO_2_ signal was observed between the two groups in the MSOT images. The clear contrast provided by MSOT images between the two groups underscores the potential of MSOT imaging as a diagnostic tool for muscle‐related diseases.

### 5.2 Quantitative Analysis of Oxygen Metabolism and Collagen Content

**FIGURE 3:**
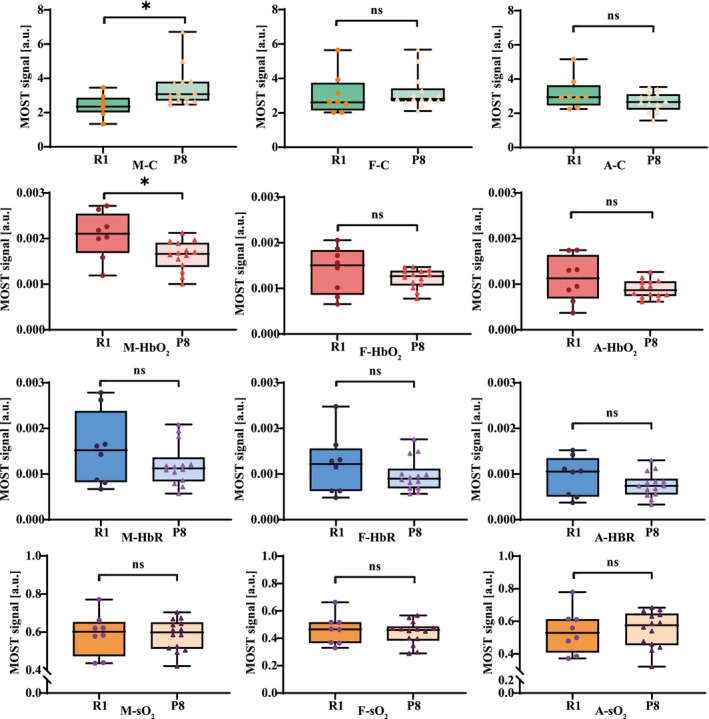
Quantitative analysis of oxygen metabolism and collagen content, displaying from top to bottom: collagen, HbO_2_, HbR and sO_2_. Column 1: muscle tissue (ROI 1), column 2: subcutaneous adipose tissue (ROI 2), column 3: femoral artery region (ROI 3). A, femoral artery; C, collagen; F, subcutaneous fat; M, muscle. *p < 0.05.

We conducted a comparative analysis of three ROI regions between R1 and P8 mice. In ROI 1 region, representing lower limb muscle tissue, P8 mice exhibited more severe muscle damage and an imbalance in collagen synthesis and degradation, resulting in dynamic changes in collagen content. At a specific stage, the collagen level in P8 mice muscles was significantly higher than in R1 mice (3.451 ± 1.159 vs. 2.409 ± 0.635 a.u., *p* = 0.030). Due to a more pronounced imbalance between oxygen demand and supply in P8 mice muscle tissue, their HbO_2_ levels were lower compared to R1 mice (0.0016 ± 0.0003 vs. 0.0021 ± 0.0005 a.u., *p* = 0.018). No significant differences in HbR and sO_2_ were observed between the two groups in the ROI 1 region. Our study revealed no significant differences in collagen, HbO_2_, HbR and sO_2_ levels in the ROI 2 and ROI 3 regions between the two groups (Figures 3) In both mouse strains, mean values for HbO_2_ and sO_2_ were significantly higher in muscular tissue compared to subcutaneous tissue. This difference was attributed to the lower vascularity and higher fat and connective tissue content in subcutaneous tissue. The results were as follows: R1‐HbO_2_ (0.0021 ± 0.0005 vs. 0.0014 ± 0.0005 a.u., *p* = 0.018), R1‐sO_2_ (0.5889 ± 0.1110 vs. 0.4486 ± 0.1256 a.u., *p* = 0.035), P8‐HbO_2_ (0.0016 ± 0.0003 vs. 0.0012 ± 0.0002 a.u., *p* = 0.001) and P8‐sO_2_ (0.5845 ± 0.0817 vs. 0.4639 ± 0.0829 a.u., *P* < 0.001). No significant differences were observed in collagen and HbR content between the muscle and subcutaneous tissues (Table 1, Supplementary Figure 4).

**TABLE 1:** MSOT mean values (a.u.) for muscular and subcutaneous tissue comparing both thighs.

		Muscle		Subcutaneous tissue		*p*
		Mean	SD	Mean	SD	
R1 left thighs (*n* = 8)	HbO_2_	0.0021	0.0005	0.0014	0.0005	0.018
	HbR	0.0016	0.0008	0.0012	0.0007	0.348
	sO_2_	0.5889	0.1110	0.4486	0.1256	0.035
	Collagen	2.4089	0.6352	2.8115	1.4915	0.494
P8 left thighs (*n* = 14)	HbO_2_	0.0016	0.0003	0.0012	0.0002	0.001
	HbR	0.0012	0.0005	0.0010	0.0004	0.162
	sO_2_	0.5845	0.0817	0.4639	0.0829	<0.001
	Collagen	3.4514	1.1593	3.2034	0.9917	0.548

### 5.3 Cross‐Validation of Models

**FIGURE 4:**
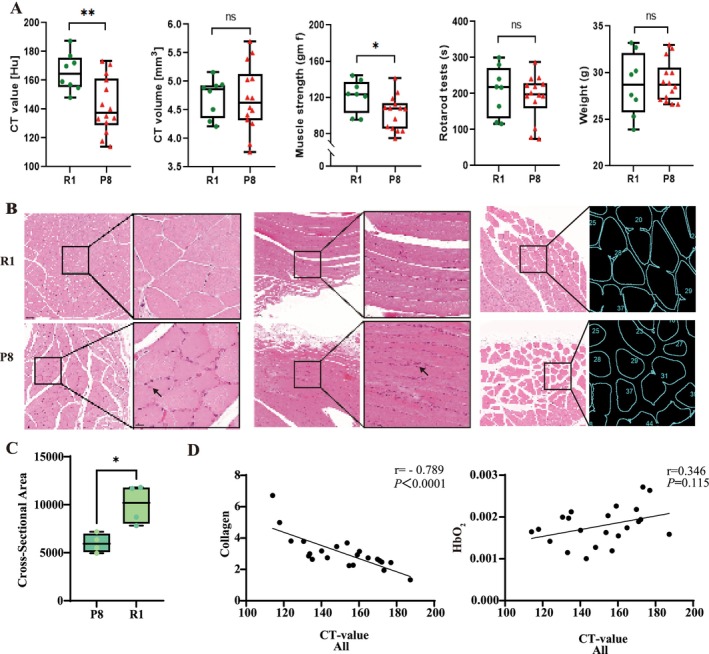
A comparison of CT, muscle strength, rotarod test and weight between P8 and R1 mice. B. Representative H&E‐stained sections: transverse views in the left column and longitudinal vies in the right column. Insets show enlargements of the boxed areas. Compared with R1 mice, P8 muscles exhibit attenuated sarcomeric striations, reduced fibre calibre and numerous centrally or eccentrically located nuclei (black arrows), whereas R1 muscles display orderly fibres with peripherally positioned nuclei. Semiautomated segmentation of transverse H&E images performed in ImageJ. The contours outline individual myofibres for subsequent morphometric analysis. C. Quantication of mean myofibre CSA per animal (R1, *n* = 4; P8, *n* = 4) Box‐and‐whisker plots show the median, inter‐quartile range and 5%–95% percentiles; by unpaired, Student's *t* test. Scale bars: 100 m (overview images) and 20 μ m (insets). D. Correlation between HbO_2_, collagen and CT value. CSA, cross‐sectional area. *p < 0.05.

To complement the MSOT results, we performed Micro‐CT scans to assess muscle structural integrity. CT values of P8 mice were significantly lower than those of R1 mice (142.5033 ± 19.272 vs. 165.659 ± 13.095 HU, *p* = 0.007) (Figure 4A), but there was although no significant difference in muscle volume was observed between the two groups at the same site. Muscle strength was significantly decreased in P8 mice in comparison to R1 mice (173.144 ± 57.798 vs. 236.126 ± 57.257 gf, *p* = 0.023). No significant differences were found in the body weight and latency‐to‐fall time.

In the H&E‐stained sections (Figure 4B), R1 muscles exhibited sharp sarcomeric striations with nuclei confined to the fibre periphery, whereas P8 muscles showed markedly attenuated striations, swollen myofibres, disordered and proliferative nuclei, many of which had migrated to eccentric positions (black arrows in the insets), indicating pronounced structural degeneration. After semiautomated segmentation of the transverse H&E images in ImageJ, morphometric analysis revealed that the mean myofibre CSA for each animal was significantly smaller in P8 mice than in R1 controls (unpaired *t* test, *p* < 0.05; Figure 4C).

**FIGURE 5:**
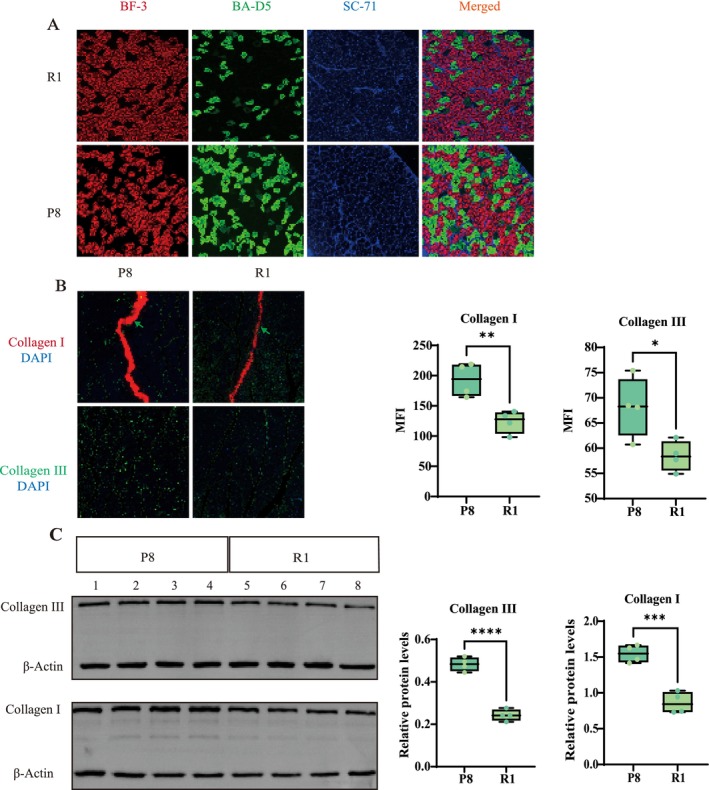
A. MHC immunofluorescence stating showed that from left to right, there were type IIb fibres (red), type I fibres (green), type IIa fibres (blue) and merged images. B. Immunofluorescence staining; collagen I (red) and collagen II (green) in the gastrocnemius muscle of P8 and R1 mice. Nuclei were counter‐stained with DAPI (blue). Green arrows indicate areas of collagen deposition. Box‐and‐whisker plots on the right quantify MFI for each group. Scale bars: 50 um for panels. C. Western lot analysis Upper panel, both normalized to β‐actin. Box‐and‐whisker plots on the right present band‐density‐normalized relative protein levels. (*n* = 4 mice per group, Student's *t* test). MFI, mean fluorescence intensity. **** *p* < 0.0001; ***p < 0.001; **p < 0.01; *p < 0.05. Error bars represent mean ± SD.

MHC immunofluorescence staining showed an increased percentage area of type I fibres (green) in the P8 group, possibly indicating a relative proportional increase or a compensatory muscle adaptation for lost strength and function. Type IIb fibres (red) were significantly reduced compared to the R1 group, correlating with the decline in muscle mass and strength. Type IIa fibres (blue) appeared to maintain relative equilibrium compared to the other fibre types, potentially indicating advanced muscle dysfunction. Closer examination revealed subtle yet significant disarray within the IIb and IIa fibre structures of P8 mice, with increased discontinuity and architectural chaos, suggesting a deeper level of muscular degeneration beyond quantitative assessments (Figure 5A).

We performed quantitative immunofluorescence and Western blot (WB) analyses to assess collagen accumulation in the gastrocnemius muscles of senescence‐accelerated P8 mice and age‐matched R1 controls. Immunofluorescence staining showed a pronounced increase in collagen III signal in P8 sections, with MFI higher than in R1 animals (*p* < 0.05; Figure 5B); collagen I also exhibited an upward trend. (*p* < 0.01; Figure 5B). Consistent with a fibrotic phenotype, collagen III fluorescence was broadly distributed throughout both the perimysium and endomysium, indicating extensive extracellular‐matrix (ECM) remodelling. WB analysis corroborated these observations: After normalization to β‐Actin, collagen III protein levels in P8 muscle were markedly greater than those in R1 (*p* < 0.0001), and collagen I expression was also significantly elevated (*p* < 0.001) (Figure 5C). Collectively, these data demonstrate that senescence‐accelerated P8 mice accumulate excessive collage, particularly the fibrosis‐associated collagen III and collagen I, signifying progressive fibrotic remodelling of skeletal muscle.

### 5.4 Correlation Between HbO_2_, Collagen and CT Value

Our investigation revealed significant differences in HbO2 levels, collagen content and CT values within the muscular tissues of R1 and P8 rodent strains. Notably, we observed a substantial inverse relationship between collagen content and CT values (*r* = −0.789, *P*<0.0001), which proved crucial in the diagnostic and evaluative framework for sarcopenia. When analyzed separately, this correlation remained significant in P8 mice (*r* = −0.676, *P* = ‐0.001) but not in R1 mice (*r* = −0.676, *P* = −0.001), likely due to limited sample size (Supplementary Figure 5). These results suggesting that collagen content may be a key determinant of muscle density.

Interestingly, our analysis did not reveal any significant correlation between muscle HbO_2_ levels and CT values, indicating that MSOT may have the capability to detect oxygen metabolism information not directly reflected in CT density measurement. MSOT could provide complementary insights into muscle health that extend beyond the scope of traditional density‐based imaging assessments (Figure 4D, Supplementary Figure 5).

## 6 Discussion

In this pilot study, we demonstrate that label‐free muscle optoacoustic imaging provides direct insights into muscle structure and metabolism. We present the first MSOT data comparing collagen, HbO_2_, HbR and sO_2_ signals in the lower limb muscles, subcutaneous fat and arterial regions between two distinct mouse models of sarcopenia: P8 and R1. P8 muscles showed lower HbO_2_ levels and reduced metabolic activity compared to healthy controls (R1). The P8 group showed significantly higher collagen content and more disordered structure, suggesting increased fibrosis—a hallmark of muscle degeneration [27, S13].

Compared to subcutaneous fat, muscles displayed stronger HbO_2_ and sO_2_ signals, likely due to differences in tissue structure and perfusion levels. Interestingly, CT values correlated with collagen signals but not with HbO_2_. This finding suggests that collagen content may be a key factor influencing muscle density. Additionally, it implies that HbO_2_ concentration in muscle tissue could serve as an alternative biomarker, reflecting aspects of muscle nutrition and metabolic health not directly captured by muscle density quantification. These observations highlight the complex nature of muscle physiology and emphasize the need for a multifaceted approach to understanding muscle health. Our study underscores the potential of MSOT as a valuable tool for the comprehensive assessment of muscle condition, offering complementary information to traditional imaging techniques.

Utilizing MSOT technology, we observed an elevated presence of collagen within the skeletal muscles of sarcopenic mice, with our results demonstrating an inverse correlation between collagen content and CT values. This correlation may be attributed to several factors. First, the sarcopenic mice exhibit significant alterations in muscle structure. Fibroblasts, the primary producers of ECM proteins in skeletal muscle, play a pivotal role in providing structural support for muscle tissue. Skeletal muscle fibrosis is characterized by disproportionate accumulation of ECM components [28]. During this process, there is an increased abundance of collagen‐producing cells, and the collagen network undergoes significant alterations [29]. Collagen, a key component of the ECM in muscle cells, accumulates abnormally and may be linked to the disease's pathogenesis [30]. There is a decrease in the production of muscle fibres, particularly the type II fibres, possibly stemming from aberrant intracellular conditions [31, S14]. Second, the development of mitochondrial insufficiency in myogenic muscle is proposed to release myokines, including fibroblast growth factor 21 (FGF21) and growth and differentiation factor 15 (GDF15). This mediates a systemic response influencing the composition of the ECM within muscle tissues [32]. Third, sarcopenia is characterized not only by muscle fibre depletion, fibrosis and elevated collagen levels but also often by fat infiltration within skeletal muscle [33, S15, S16]. Research has shown a negative correlation between the quantity of intramuscular adipose tissue and CT values [34]. The inverse association observed between collagen content and CT values aligns with these multifaceted changes in muscle composition and structure associated with sarcopenia.

Muscle blood perfusion is a crucial metric that significantly influences muscle function, directly correlating with both intramuscular HbO_2_ and HbR levels [18, S17]. MSOT distinguishes itself by providing a comprehensive assessment of intramuscular circulation in a single, noninvasive examination. It offers a label‐free, continuous monitoring approach, allowing for precise detection of HbO_2_ and Hb levels [18, 20, S18, S19]. In our study, we observed changes in HbO_2_ levels within the microcirculation of sarcopenic mice at rest, without any manipulation or vasodilation [18, 19], while sO_2_ and HbR remained relatively stable. Previous research has suggested that a sparse capillary network precedes sarcopenia, potentially leading to a reduced HbO_2_. While capillaries are crucial for oxygen delivery, capillary supply to muscle fibres relates more to fibre size than oxidative capacity [35, S17]. This phenomenon may indicate that HbO_2_ is a more sensitive metabolic indicator for sarcopenia, although this slight change was insufficient to affect overall oxygen saturation.

The observed decrease in HbO_2_ levels in sarcopenia may have profound implications for muscle function. Reduced oxygenated haemoglobin levels likely reflect decreased oxygen supply to muscle tissue, potentially leading to mitochondrial dysfunction and reduced ATP production [36, 37]. Chronic oxygen deficiency may trigger adaptive responses, such as muscle fibre type conversion (from fast‐twitch to slow‐twitch fibres), thereby affecting muscle contraction speed and strength [38]. Insufficient oxygen supply exacerbates oxidative stress, promotes muscle protein degradation and apoptosis, and accelerates muscle loss, [39] making HbO_2_ level monitoring a critical indicator for assessing sarcopenia progression and treatment efficacy, while conventional CT and MRI lack real‐time dynamic monitoring capability.

The diminished levels of HbO_2_ in sarcopenia may be linked to several factors, including the proliferation of reactive oxygen species (ROS) [40], declined mitochondrial capacity [41], modifications in cytochrome c oxidase (CCO) [42] and/or inflammation within muscle tissues [43]. Further validation and extensive research are necessary to better understand these underlying factors and mechanisms. We also discovered that the HbO_2_ and sO_2_ levels in subcutaneous tissue of both sarcopenic and control mice were lower than in the muscle tissue, aligning with the findings reported by Helfen et al. [26] The observed increase in muscle microcirculation in sarcopenic mice could be due to more abundant tissue perfusion, larger‐diameter intramuscular blood vessels or a richer capillary network [26, S17, S20]. Notably, as the depth of noise reduction in the deep muscular tissues increases, neither light scattering nor signal attenuation impacts our outcomes, highlighting the distinctive benefits of MSOT technology in the study of muscle tissue. Finally, we found no significant correlation between muscle HbO_2_ and CT values. This finding aligns with other research, indicating that the oxidative capacity of skeletal muscle determines its metabolic capability and maintains muscle performance [44]. This may be associated with the capacity of mitochondria within the muscle to produce energy, rather than being directly linked to the processes affecting oxygen metabolism [44, 45].

P8 mouse is the most commonly used accelerated aging mouse model in sarcopenia research, with these mice already exhibiting early stages of sarcopenia at 8 months [1]. To ensure the reliability of our model, we conducted comprehensive cross‐validation from multiple aspects, including behaviour, muscle structure, pathology and immunofluorescence. Muscle strength is currently considered the most reliable measurement index for sarcopenia. Our primary choice was the noninvasive measurement of forelimb gripping force, which can assess the maximum contraction force of the forelimb during autonomous activity [1, S21]. Our findings aligned with numerous previous studies on sarcopenia mice, demonstrating a decrease in muscle strength of P8 mice decreased during this period [21, 36, S22, S23].

CT values are widely regarded as the gold standard for evaluating quantitative and qualitative changes in skeletal muscle mass. Higher CT values indicate greater skeletal muscle density [6, S7, S23]. Our research revealed a discernible disparity, with P8 mice exhibiting less optimal muscle mass compared to their R1 counterparts.

To further enrich our understanding, we examined the microscopic architecture of our chosen model. Histological examination using H&E staining showed striking similarities to Zhao's findings, characterizing the presence of sarcopenia through the lens of muscle atrophy, disarray of muscle fibres, abnormal distribution of cell nuclei and diminished CSA of the muscle tissue [21].

Immunofluorescence staining demonstrated a reduction in both collagen I and III in P8 mouse muscle compared with R1 controls and revealed subtle cellular transitions in the muscle regeneration process of sarcopenic mice. As muscle fibres undergo senescence, we observed a notable decline in type II fibres, with a subset of these fibres transforming into type I fibres. This transition consequently led to a proportional increase in the prevalence of type I fibres [31, 38]. After comprehensive consideration of these findings, we are confident that our chosen model serves as a reliable framework for exploring sarcopenia. The consistency of our results with established literature and the multifaceted approach to model validation reinforce the robustness of our experimental design and the validity of our conclusions.

CT and MRI remain gold standards for structural assessment of muscle mass and fat infiltration but lack dynamic real‐time monitoring of muscular oxygen metabolism and microvascular oxygen flux during contraction [12, 13]. They cannot detect early hypoxia signatures or monitor metabolic changes. The introduction of MSOT technology could significantly alter current strategies for diagnosing and managing sarcopenia. First, the real‐time, multiparameter assessment provided by MSOT may enable early diagnosis of sarcopenia, potentially even before traditional muscle mass and strength indicators show significant changes. This could create opportunities for early intervention, potentially greatly improving prognosis. Second, the noninvasive nature and repeatability of MSOT allow for frequent assessments, which can be used to precisely monitor disease progression and treatment response. Third, the detailed metabolic information provided by MSOT may contribute to the development of individualized treatment plans, such as adjusting exercise prescriptions or nutritional interventions based on oxygenation status. Lastly, MSOT could become a powerful tool for evaluating the efficacy of novel sarcopenia treatments, accelerating drug development and clinical trial processes.

## 7 Limitations

Despite rigorous validation of our model and adherence to stringent experimental standards, our study has several limitations. First, while MSOT data were collected from awaken mice to minimize anaesthesia‐induced effects on muscle microcirculation and oxygen metabolism, the ankle restraint method could potentially influence venous return in the lower limbs; future studies should employ pressure‐controlled restraint to standardize immobilization and mitigate this effect. Second, although proximal muscle regions are presumed to have higher collagen content, quantifying total collagen alone may be insufficient; future MSOT muscle studies would benefit from algorithm refinement and enhanced image segmentation to better delineate internal composition, considering both collagen variation and connective tissue influences. Third, using presarcopenic P8 mice without pharmacological or exercise interventions might not fully capture sarcopenia model characteristics, while this approach indirectly demonstrates MSOT's capability for early muscle abnormality detection akin to CT; stronger theoretical support in future sarcopenia‐related MSOT research could derive from models exhibiting more pronounced muscle abnormalities. Fourth, the relatively small scale and cross‐sectional nature of our study limit generalizability, and the current restriction of MSOT to animal models, alongside a lack of clinical implementation data, may constrain translational impact. Fifth, while pathological specimens validated collagen content, they cannot reflect in vivo muscle oxygen metabolism; our study lacked cross‐validation using live techniques (e.g., near‐infrared spectroscopy, fluorescent probes). We are actively exploring clinical applications of MSOT to address this limitation.

## 8 Conclusions

This study presents a novel approach to investigate oxygen metabolism and collagen distribution in sarcopenic skeletal muscle using MSOT technology. The application of MSOT is noninvasive, highly efficient and convenient, allowing for real‐time monitoring of local muscle indicators and enabling comparative and analytical studies of oxygen metabolism in different regions of sarcopenic mouse muscles. Our findings also reveal a correlation between muscle collagen content and CT values, which is significant given that CT is the most established detection method for sarcopenia. This finding suggests that MSOT could provide complementary information to traditional imaging techniques in assessing muscle health, particularly in detecting metabolic changes that may precede structural alterations visible on CT.

While acknowledging the limitations of our study, such as the use of a pre‐sarcopenia mouse model and the need for further validation in human subjects, the potential of MSOT in sarcopenia research is promising. Looking ahead, MSOT technology holds promise for facilitating valuable research on various aspects of sarcopenia, including muscle morphology, function and oxygen metabolism. Future studies can build upon these findings to further refine and validate the use of MSOT in sarcopenia diagnosis and treatment monitoring.

## Author Contributions

YY completed the preliminary concept, conducted experimental design and implementation, analyzed the data, generated the figures, conducted the literature search, and wrote the manuscript. DW and SXJ contributed to method conceptualization and completed MSOT data reconstruction. YQX, YHX and YYB assisted in completing MSOT data collection. JZ and YW developed the statistical approach and contributed to data interpretation. TMP and ZL oversaw data quality control. YRZ assisted in collecting H&E histology data. JHC assisted in collecting CT data. XYC and BLW assisted in collecting immunohistochemistry data. SPC revised the manuscript. MY and HBJ oversaw overall study design and general implementation, and revised the manuscript. All authors contributed to the conception or design of the work or the acquisition of data for the work. All authors reviewed and approved the final manuscript and agree to be accountable for all aspects of the work.

## Conflicts of Interest

The authors declare no conflicts of interest.

## Supporting information


**Figure S1:** jcsm70088‐sup‐0001‐Supplementary_Material.docx. Selection of measured parameters based on multiwavelength algorithm.
**Figure S2:** Schematic diagram of multi wavelength imaging method.
**Figure S3:** The method for selecting the ROI of micro‐CT.
**Figure S4:** Immunofluorescence analysis of collagen distribution in P8 and R1 mice revealed comparable collagen levels between muscle and subcutaneous tissues.
**Figure S5:** The correlation between HbO_2_, collagen and CT values in different mouse species.

**Data S1:** Supplementary Information.
