# Supplementary material for: Multispectral Optoacoustic Tomography of Skeletal Muscle Unveils Microcirculation and Oxygen Metabolism Alterations in Sarcopenia
Source: J Cachexia Sarcopenia Muscle. 2025 Oct 21;16(5):e70088. doi: 10.1002/jcsm.70088 (PMC12538309; doi:10.1002/jcsm.70088)
Supplement: Supplementary file 1 — Figure S1: jcsm70088‐sup‐0001‐Supplementary_Material.docx. Selection of measured parameters based on multiwavelength algorithm. Figure S2: Schematic diagram of multi wavelength imaging method. Figure S3: The method for selecting the ROI of micro‐CT. Figure S4: Immunofluorescence analysis of collagen distribution in P8 and R1 mice revealed comparable collagen levels between muscle and subcutaneous tissues. Figure S5: The correlation between HbO2, collagen and CT values in different mouse species. [file JCSM-16-e70088-s001.docx]

**Supplementary materials**

**Supplementary Figure 1.** Selection of measured parameters based on

multi-wavelength algorithm

**Supplementary Figure 2** Schematic diagram of multi wavelength imaging method

**Supplementary Figure 3.** The method for selecting the ROI of Micro-CT

**Supplementary Figure 4.** Immunofluorescence analysis of collagen distribution

in P8 and R1 mice revealed comparable collagen levels

between muscle and subcutaneous tissues

**Supplementary Figure 5.** The correlation between HbO_2_, collagen and CT values

in different mouse species

**Supplementary Explanation 1.** Basic concepts and fundamental principles of

multi-wavelength imaging calculations

**Supplementary Explanation 2.** Explanation of arbitrary units (a.u.) in multispectral

optoacoustic tomography (MSOT)

**Supplementary Figure 1.** Selection of measured parameters based on

multi-wavelength algorithm


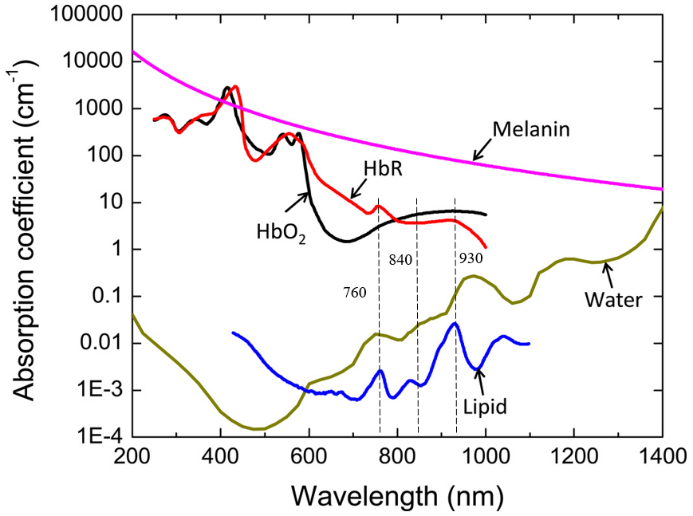


The wavelength-dependent optical absorption coefficients of major chromophores in biological tissue are shown in Supplementary Figure 1 (above). As evident from the figure, deoxyhemoglobin (HbR) exhibits a higher absorption coefficient than oxyhemoglobin (HbO_2_) within the wavelength range of 600 nm to 790 nm. A distinct absorption peak for HbR occurs at 760 nm, where a significant difference exists between the absorption coefficients of HbR and HbO_2_ . Conversely, HbR displays a local absorption minimum (valley) at 840 nm, at which point another significant difference between the absorption coefficients of HbR and HbO_2_ is observed.

﻿

Consequently, 760 nm and 840 nm correspond to the absorption peaks of HbR and HbO_2_, respectively. At these specific wavelengths, the concentration of each hemoglobin species exerts its most pronounced effect on the photoacoustic signal. It is precisely because there are the most significant differences between the two substances in these two bands that we can easily quantify the relative content of the two substances in the substance.Therefore, the quantitative data for HbR and HbO_2_ calculated from photoacoustic images acquired at these wavelengths are inherently more accurate. ^[1, 2, 3]^ (Supplementary Explanation 1)

﻿

Collagen has strong absorption around 930-1000 nm in the infrared. Our laser's standard mode (680-960 nm) can't reach 1000nm, requiring a different mode (OPO) which takes about 10 seconds to switch to.

For accurate measurements, multispectral photoacoustic data needs to be taken with 1 second between different wavelengths. Considering the need for fast data collection and stability of laser energy, we chose 930 nm (within the standard mode range) for detecting collagen. ^[3, 4]^

**Supplementary Figure 2.** Schematic diagram of multi wavelength imaging method


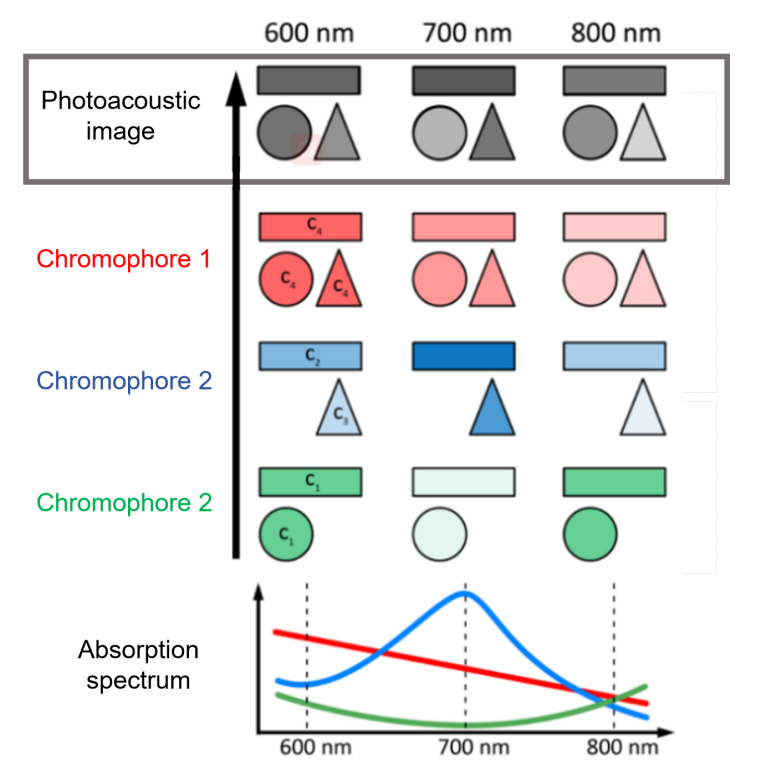


As shown in Supplementary Figure 2, suppose there are three materials with different structures (represented by circles, rectangles, and triangles, respectively). Each material contains three chromophores (C1, C2, C3) at different concentrations. According to the extended application of the Beer - Lambert Law, the total absorption coefficient of a material at a specific wavelength is the sum of the products of the absorption coefficients and relative concentrations of each chromophore in the material at that wavelength. Thus, to determine the relative concentrations of the chromophores in a material, an n - variable linear equation system (n is the number of chromophore types) needs to be established based on the photoacoustic signals (or absorption data) of the material measured at n different wavelengths. Then, solving this system yields the relative concentration distribution. ^[4]^

**Supplementary Figure 3.** The method for selecting the ROI of Micro-CT


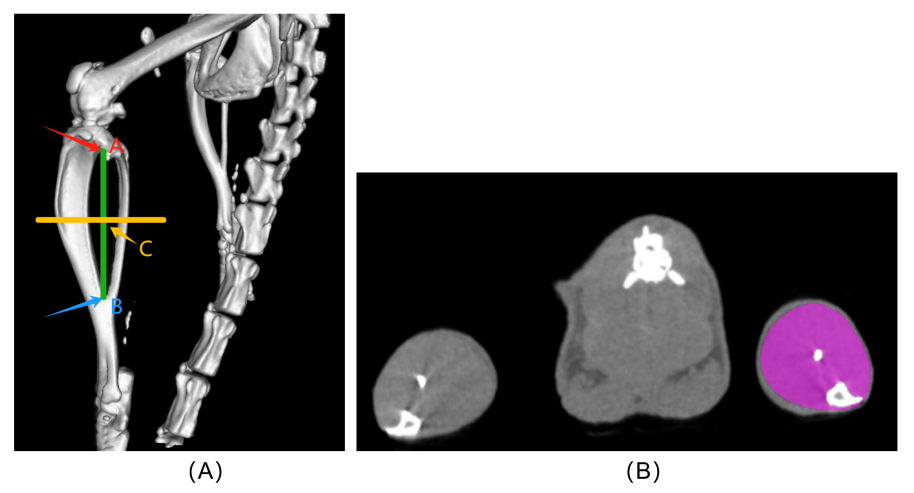


The method for selecting the ROI of Micro-CT involves the following steps: First, by using cross positioning, determine the upper intersection point A and lower intersection point B of the tibia and fibula for each mouse. Then, take the midpoint C of the line connecting points A and B as the target point. Next, select the plane that passes through point C and is perpendicular to line AB. This plane is considered to represent the maximum cross-sectional area of the tibia. To reduce errors, draw a cross-sectional muscle ROI with a distance of 1 mm above and below each layer. Calculate the density for each single layer and then determine the average density. Finally, obtain the ROI volume within a height of 2 cm in this area. Note that the term 'tibialis muscle' may be used instead of 'tibial cross-sectional muscle' for clarity. Figures (A) and (B) illustrate these steps.

**Supplementary Figure 4.** Immunofluorescence analysis of collagen distribution

in P8 and R1 mice revealed comparable collagen levels between muscle and subcutaneous tissues

**
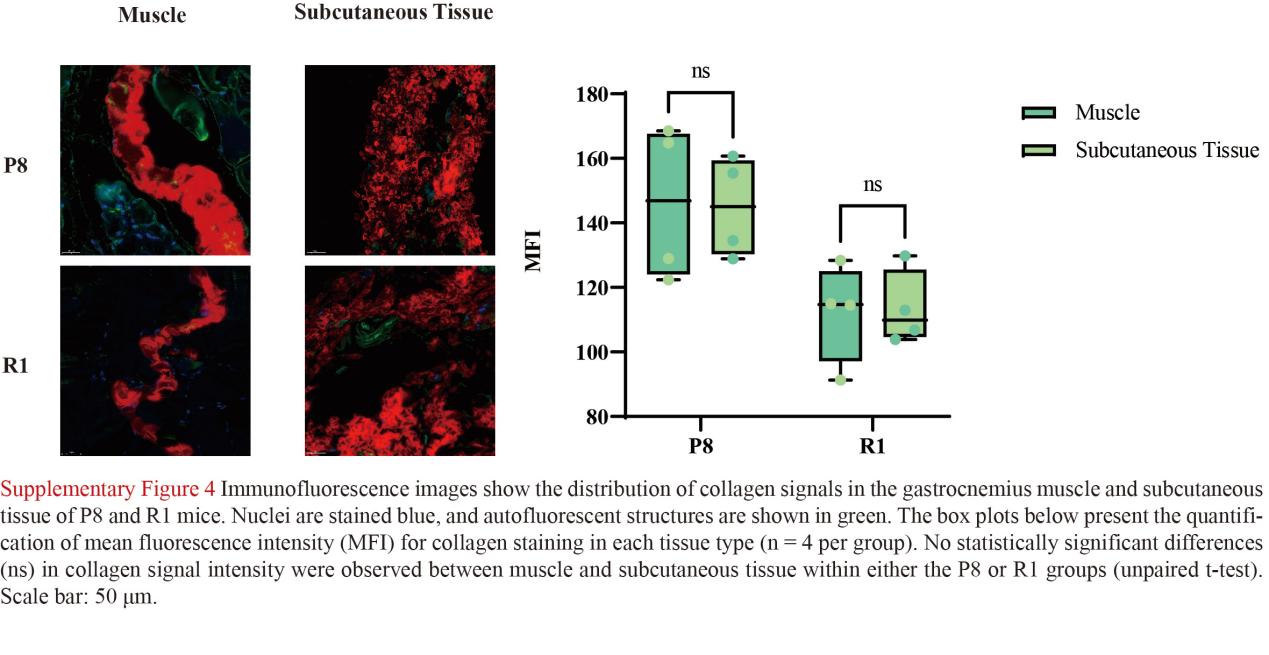
**

**Supplementary Figure 4.** The correlation between HbO_2_, collagen and CT values

in different mouse species

**
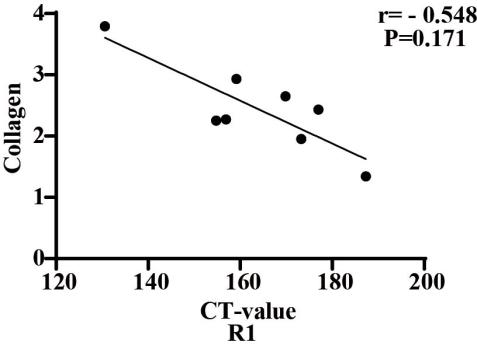
**

**
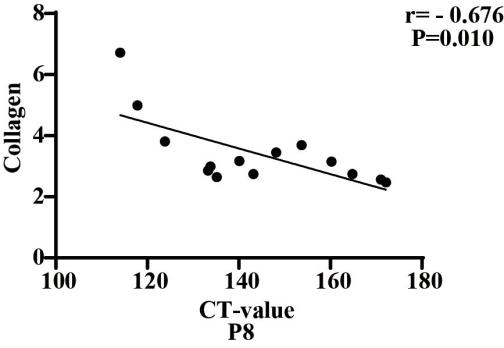
**

**
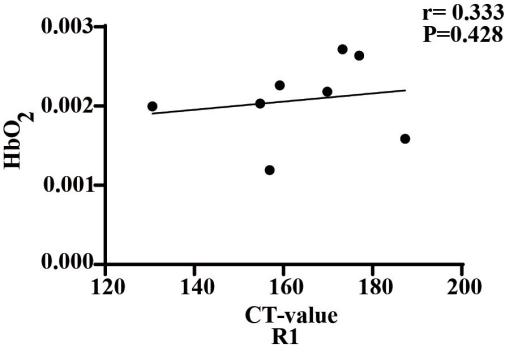
**

**
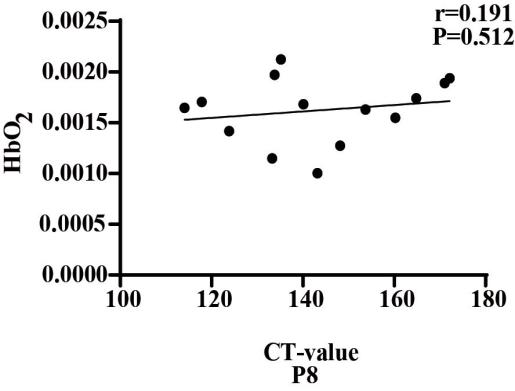
**

**Supplementary Explanation 1.** Basic concepts and fundamental principles of

multi-wavelength imaging calculations

**Fundamental Concept of Multispectral Imaging**

In practical biological tissue applications, due to the large number of chromophores in tissues and the weak absorption of most, concentration quantitative analysis usually focuses on major chromophores with significant laser absorption, such as oxyhemoglobin (HbO₂), deoxy-hemoglobin (HbR), water, collagen, and melanin. The specific selection depends on the target tissue type.

In this study, the research subject is mouse leg muscle tissue. In this tissue, HbO₂, Hb, and collagen are the dominant absorbing chromophores, with other chromophores' contributions being relatively negligible. Therefore, we target the concentration changes of HbO₂, Hb, and collagen for quantitative analysis. Using the molar (or specific) absorption coefficients of these three chromophores at 760 nm, 840 nm, and 930 nm provided in Figure 2, we construct a three - variable linear equation system based on the photoacoustic signals (or absorption values) at these three wavelengths. Finally, we solve this system to obtain the relative concentration distribution of these three chromophores in mouse leg muscle tissue.

**Multispectral Unmixing Methodology**

Following Supplementary Figure 1, our linear unmixing algorithm utilizes PA data acquired at 760 nm, 840 nm, and 930 nm. Absorption coefficients of HbO₂, HbR, and collagen at these wavelengths are incorporated into the

endogenous contrast model:

$$\mu_{a}(\bar{r},\lambda)=\varepsilon_{HbR}(\lambda)[HbR](\bar{r})+\varepsilon_{HbO}(\lambda)[HbO](\bar{r})+\varepsilon_{\text{Collagen}}(\lambda)[\text{Collagen}](\bar{r})$$

$$sO_{2}=\frac{[HbO]}{[HbT]}$$

In the formula, $\mu_{a}(\bar{r},\lambda)$represents the PA signal intensity at position$\vec{r}$ on the PA image at wavelength $\lambda$; $[HbO]$, $[Hb]$, and $[\text{Collagen}]$ respectively represent the concentration distributions of HbO_2_, HbR, and collagen at wavelength $\lambda$; $\varepsilon_{HbO_{2}}$, $\varepsilon_{Hb}$, and $\varepsilon_{\text{Collagen}}$ are the absorption coefficients of HbO_2_, HbR, and collagen at $\lambda$. During specific computations, the matrix operation is simplified to:

​​

Where

$$\mu_{a}(\overrightarrow{r})=\left[\begin{matrix} \mu_{a}(\overrightarrow{r},760) \\ \mu_{a}(\overrightarrow{r},840) \\ \mu_{a}(\overrightarrow{r},930) \end{matrix}\right]$$

$$\varepsilon(\lambda)=\left[\begin{matrix} \varepsilon_{HbO_{2}}(760) & \varepsilon_{HbR}(760) & \varepsilon_{collagen}(760) \\ \varepsilon_{HbO_{2}}(840) & \varepsilon_{HbR}(840) & \varepsilon_{collagen}(840) \\ \varepsilon_{HbO_{2}}(930) & \varepsilon_{HbR}(930) & \varepsilon_{collagen}(930) \end{matrix}\right]$$

​Through matrix transformation, we can obtain:

**Physiological Significance**

This algorithm quantifies spatial distributions of:

Oxygenated hemoglobin (HbO_2_)

Deoxygenated hemoglobin (HbR)

Collagen

Oxygen saturation (sO_2_ = [HbO_2_]/([HbO_2_]+[HbR]))

Mapping these hemodynamic parameters establishes correlations between PA signatures and pathological changes. This quantitative approach enhances disease diagnosis, treatment monitoring, and pathological assessment by objectively characterizing lesion severity.^[4-6]^

**Supplementary Explanation 2.** Explanation of arbitrary units (a.u.) in multispectral optoacoustic tomography (MSOT)

The designation "a.u." (arbitrary units) denotes relative quantitative values of hemodynamic parameters (e.g., hemoglobin concentration, oxygen saturation-derived metrics) in photoacoustic imaging, rather than absolute physical units. This convention arises from three key considerations:

**Algorithm-Dependent Relative Quantification**

Hemodynamic parameters derived through multispectral quantitative photoacoustic imaging (qPAI) algorithms inherently represent relative concentration distributions. These values are calculated via spectral unmixing of photoacoustic signal amplitudes and optical inversion solvers. However, absolute quantification at single timepoints remains constrained by biological variables (e.g., spatial heterogeneity in optical scattering/absorption) and instrumental factors (e.g., source energy fluctuations).

**Clinical Relevance and Analytical Utility**

The clinical significance of this approach lies in identifying comparative differences between pathological and normal tissues (e.g., tumor vs. periphery) or temporal hemodynamic changes (e.g., oxygenation dynamics during ischemia-reperfusion). Such analyses rely on relative trends rather than absolute values, rendering "a.u." appropriate for cross-sectional or longitudinal comparisons without requiring equivalence to clinical units (e.g., g/dL).

**Algorithmic Advancements Toward Absolute Quantification**

Emerging absolute quantification methodologies progressively narrow the gap between optoacoustic measurements and clinically standardized units. Continuous refinements in algorithms—including deep learning-based spectral calibration models, multimodal fusion frameworks, dynamic hemodynamic modeling, and Hybrid Spectral Modeling—collectively enhance interpretability of MSOT-derived data, enabling clinicians to extract clinically actionable insights with greater translational relevance.

**Conclusion**

The use of "a.u." reflects the current transitional phase in quantitative photoacoustic imaging within complex biological systems. Its scientific validity stems from enabling comparable physiological/pathological dynamics. Ongoing algorithmic validation—through ex vivo blood phantom calibrations and clinical gold-standard cross-verification—promises future convergence with standardized clinical units.

**References**

^[1]^ Jinge Yang. Research on fast photoacoustic imaging system and its applications [PhD dissertation, University of Electronic Science and Technology of China] (2019)

^[2]^ Altaf Hussain, Wilma Petersen, Jacob Staley, Erwin Hondebrink, and Wiendelt Steenbergen, "Quantitative blood oxygen saturation imaging using combined photoacoustics and acousto-optics," Opt. Lett. 41, 1720-1723 (2016)

^[3]^ Laufer, Jan; Delpy, Dave; Elwell, Clare; Beard, Paul . Quantitative spatially resolved measurement of tissue chromophore concentrations using photoacoustic spectroscopy: application to the measurement of blood oxygenation and haemoglobin concentration. Physics in Medicine and Biology, 52(1), 141–168. (2007)

^[4]^ H. B. Jiang. Photoacoustic tomography[M]. CRC Press, (2014).

^[5]^ BEARD P. Biomedical photoacoustic imaging[J]. Interface Focus, 1(4): 602-631.(2007).

^[6]^ Taehoon, Bok,Eno, Hysi,Michael C, Kolios,Quantitative ultrasound and photoacoustic assessments of red blood cell aggregation in the human radial artery.[J] .Photoacoustics, 43: 0.（2025）
