# Supplementary material for: Multispectral Optoacoustic Tomography of Skeletal Muscle Unveils Microcirculation and Oxygen Metabolism Alterations in Sarcopenia
Source: J Cachexia Sarcopenia Muscle. 2025 Oct 21;16(5):e70088. doi: 10.1002/jcsm.70088 (PMC12538309; doi:10.1002/jcsm.70088)
Supplement: Supplementary file 2 — Data S1: Supplementary Information. [file JCSM-16-e70088-s002.docx]

**Supplementary references**

S1. Cruz-Jentoft AJ, Bahat G, Bauer J, Boirie Y, Bruyère O, Cederholm T, et al. Sarcopenia: revised Euronpean consensus on definition and diagnosis. Age and ageing. 2019 Jan 1;48(1):16-31.

S2 Sousa-Santos AR, Barros D, Montanha TL, Carvalho J, Amaral TF. Which is the best alternative to estimate muscle mass for sarcopenia diagnosis when DXA is unavailable? PloS one. 2021 Nov-Dec;97:104517.

S3. Picca A, Calvani R. Molecular Mechanism and Pathogenesis of Sarcopenia: An Overview. 2021 Mar 16;22(6).

S4. Lopes KG, Farinatti P, Bottino DA, de Souza M, Maranhão PA, Bouskela E, et al. Sarcopenia in the elderly versus microcirculation, inflammation status, and oxidative stress: A cross-sectional study. Clinical hemorheology and microcirculation. 2022;80(2):185-95.

S5 Wen Y, Wu D, Zhang J, Jiang S, Xiong C, Guo D, et al. Evaluation of Tracheal Stenosis in Rabbits Using Multispectral Optoacoustic Tomography. Frontiers in bioengineering and biotechnology. 2022;10:860305.

S6.  Ranjbar H, Soti M, Janahmadi M, Kohlmeier KA, Sheibani V, Ahmadi-Zeidabadi M, et al. Modulation of the CB1 cannabinoid receptor has potential therapeutic utility in the 3-acetylpyridine cerebellar ataxia rat model. Experimental Brain Research. 2022;240(9):2339-48.

S7. Wang J, Cui C, Chim YN, Yao H, Shi L, Xu J, et al. Vibration and β-hydroxy-β-methylbutyrate treatment suppresses intramuscular fat infiltration and adipogenic differentiation in sarcopenic mice. 2020 Apr;11(2):564-77.

S8 Shang G, Han L, Wang Z, et al. Sarcopenia is attenuated by TRB3 knockout in aging mice via the alleviation of atrophy and fibrosis of skeletal muscles. J Cachexia Sarcopenia Muscle. 2020;11(4):1104-1120. doi:10.1002/jcsm.12560

S9 Zhu GZ, Zhao K, Li HZ, et al. Melatonin ameliorates age-related sarcopenia by inhibiting fibrogenic conversion of satellite cell. Mol Med. 2024;30(1):238. doi:10.1186/s10020-024-00998-2

S10 Kim A, Park SM, Kim NS, Park M, Cha S. Ginsenoside rc prevents dexamethasone-induced muscle atrophy and enhances muscle strength and motor function. J Ginseng Res. 2025;49(1):42-52. doi:10.1016/j.jgr.

S11 Kanazawa Y, Miyachi R, Higuchi T, Sato H. Effects of aging on collagen in the skeletal muscle of mice. Int J Mol Sci. 2023;24(17):13121. doi:10.3390/ijms241713121

S12 Crowe AR, Yue W. Semi-quantitative determination of protein expression UsingImmunohistochemistry staining and analysis. Bio-Protoc. 2019;9(24):e3465.

S13 Farup J, Just J, de Paoli F, Lin L, Jensen JB, Billeskov T, et al. Human skeletal muscle CD90+ fibro-adipogenic progenitors are associated with muscle degeneration in type 2 diabetic patients. Cell Metabolism. 2021;33(11):2201-14.e10.

S14 Carter CS, Justice JN, Thompson L. Lipotoxicity, aging, and muscle contractility: does fiber type matter? GeroScience. 2019 Jun;41(3):297-308.

S15 Kalinkovich A, Livshits G. Sarcopenic obesity or obese sarcopenia: A cross talk between age-associated adipose tissue and skeletal muscle inflammation as a main mechanism of the pathogenesis. Ageing research reviews. 2017 May;35:200-21.

S16 Li CW, Yu K. Pathogenesis of sarcopenia and the relationship with fat mass: descriptive review. 2022 Apr;13(2):781-94.

S17. Hendrickse P, Degens H. The role of the microcirculation in muscle function and plasticity. Journal of Muscle Research and Cell Motility. 2019;40(2):127-40.

S18. Li Y, Gröhl J, Haney B, Caranovic M, Lorenz-Meyer E, Papatheodorou N, et al. Teachability of multispectral optoacoustic tomography. 2024 May 8:e202400106.

S19. Nguyen VP, Henry J, Zhe J, Hu J, Wang X, Paulus YM. Multimodal imaging of laser-induced choroidal neovascularization in pigmented rabbits Scientific reports. 2023 May 24;13(1):8396.

S20. Thorn CE, Shore AC. The role of perfusion in the oxygen extraction capability of skin and skeletal muscle. American journal of physiology Heart and circulatory physiology. 2016 May 15;310(10):H1277-84.

S21.Otsuka S, Fukumaru K, Tani A, Takada S, Kikuchi K, Norimatsu K, et al. Analysis of the Effects of Ninjin'yoeito on Physical Frailty in Mice. 2022 Sep 23;23(19).

S22. Wang C, Zhao B, Zhai J, Wang A. Clinical-grade human umbilical cord-derived mesenchymal stem cells improved skeletal muscle dysfunction in age-associated sarcopenia mice. 2023 May 12;14(5):321.

S23. Selvais CM, Davis-López de Carrizosa MA, Nachit M, Versele R, Dubuisson N, Noel L, et al. AdipoRon enhances healthspan in middle-aged obese mice: striking alleviation of myosteatosis and muscle degenerative markers. 2023 Feb;14(1):464-78.

S24. Binder-Markey BI, Broda NM, Lieber RL. Intramuscular Anatomy Drives Collagen Content Variation Within and Between Muscles. Frontiers in physiology. 2020;11.
